# Silencing MicroRNA-137-3p, which Targets RUNX2 and CXCL12 Prevents Steroid-induced Osteonecrosis of the Femoral Head by Facilitating Osteogenesis and Angiogenesis

**DOI:** 10.7150/ijbs.38713

**Published:** 2020-01-14

**Authors:** Lingchi Kong, Rongtai Zuo, Mengwei Wang, Wenbo Wang, Jia Xu, Yimin Chai, Junjie Guan, Qinglin Kang

**Affiliations:** Department of Orthopedic surgery, Shanghai Jiao Tong University Affiliated Sixth People's Hospital, Shanghai 200233, P.R. China

**Keywords:** steroid-induced osteonecrosis of the femoral head, miR-137-3p, bone marrow-derived mesenchymal stem cells, CXCL12 (SDF-1α), Runx2, endothelial progenitor cells

## Abstract

The main pathogenesis of steroid-induced osteonecrosis of the femoral head (SONFH) includes decreased osteogenic capacity of bone marrow-derived mesenchymal stem cells (BMSCs) and damaged blood supply to the femoral head. MicroRNAs (miRNAs) have been shown to play prominent roles in SONFH development. However, there is no report that a specific miRNA targeting two genes in two different pathogenic pathways has been applied to this disease. The present study investigated the effects of transplantation of miR-137-3p-silenced BMSCs on the prevention and early treatment of SONFH. First, western blotting and dual luciferase assays were employed to verify that miR-137-3p directly targets Runx2 and CXCL12. Then, silencing of miR-137-3p was found to facilitate osteogenic differentiation of BMSCs, which was confirmed by alkaline phosphatase (ALP) staining, alizarin red staining and qRT-PCR. Silencing of miR-137-3p also promoted angiogenesis by human umbilical vein endothelial cells (HUVECs) in the presence or absence of glucocorticoids. Thereafter, overexpression of Runx2 and CXCL12 without the 3′ untranslated region (3′UTR) partially rescued the effects of miR-137-3p on osteogenesis and angiogenesis, respectively. This finding further supported the hypothesis that miR-137-3p exerts its functions partly by regulating the genes, Runx2 and CXCL12. We also demonstrated that SONFH was partially prevented by transplantation of miR-137-3p-silenced BMSCs into a rat model. Micro-CT and histology showed that the transplantation of miR-137-3p-silenced BMSCs significantly improved bone regeneration. Additionally, the results of enzyme-linked immunosorbent assays (ELISA) and flow cytometry suggested that stromal cell-derived factor-1α (SDF-1α) and endothelial progenitor cells (EPCs) participated in the process of vascular repair. Taken together, these findings show that silencing of miR-137-3p directly targets the genes, Runx2 and CXCL12, which can play critical roles in SONFH repair by facilitating osteogenic differentiation and mobilizing EPCs.

## Introduction

Steroid-induced osteonecrosis of the femoral head (SONFH) is a devastating orthopedic disease 1, which is characterized by glucocorticoid-induced progressive deterioration of the hip joint 2, and severely affects patients' quality of life 3. Thus far, the identified pathogenic mechanisms of this disorder include a steroid-induced decrease in the osteogenic capacity of bone marrow-derived mesenchymal stem cells (BMSCs) 4, damage to the blood supply of the femoral head 5, osteoblast apoptosis associated with endoplasmic reticulum stress 6, 7 and autophagy 8. Therefore, enhancement of osteogenesis and angiogenesis could potentially facilitate the prevention and early treatment of SONFH.

To date, BMSC transplantation has been widely applied to treat orthopedic diseases, including SONFH, but satisfactory efficacy has not always been achieved by stem cell transplantation alone 9, 10. Several studies have suggested that specifically-modified BMSCs exerted more powerful effects in SONFH. Most of the engineered genes were associated with osteogenesis and angiogenesis, such as P-glycoprotein 11, vascular endothelial growth factor (VEGF)-165 12 and fibroblast growth factor (FGF)-2 13. These genes significantly increased the repair efficiency of stem cells. Based on these results, gene-engineered stem cell transplantation is a promising strategy for the prevention and treatment of SONFH.

MicroRNAs (miRNAs) are a type of small, non-coding RNAs. MiRNAs play important roles in post-transcriptional regulation by interacting with the 3′ untranslated region (3′UTR) of target genes 14, 15. Interestingly, recent studies reported that some miRNAs were involved in the pathophysiological processes of SONFH, most of which were associated with osteogenesis 16 or angiogenesis 17. However, little research has focused on specific miRNA molecules that could enhance both osteogenesis and angiogenesis.

MicroRNA-137 (miR-137) has been reported to be associated with both alkaline phosphatase (ALP) activity 18 and angiogenesis 19. Recently, *Zhang et al.* for the first time reported that runt-related transcription factor 2 (Runx2) was a potential direct target of miR-137 20, which suggested that miR-137 may be involved in osteogenesis mediated by Runx2. However, they did not perform experiments to verify the above hypotheses. C-X-C chemokine 12 (CXCL12) is an important angiogenic factor 21, which was also previously identified as a direct target of miR-137 22. It is well known that stromal cell-derived factor-1α (SDF-1α) is the product of CXCL12. Activation of the SDF-1α/C-X-C chemokine receptor 4 (CXCR4) axis has been implicated in the process of angiogenesis, including recruitment of endothelial progenitor cells (EPCs) 23, 24 and endothelial cell migration 25. EPCs are a type of bone marrow-derived premature progenitor cells 26, which are characterized as CD45^low^/CD34^+^/VEGFR2^+^ 27. Accumulating evidence has indicated that glucocorticoid abuse reduces the quantity and quality of circulating EPCs 28, which play a significant role in vascular repair in the ischemic necrotic area of SONFH 29. Therefore, the CXCL12/CXCR4 axis and EPCs are promising therapeutic targets in SONFH. Although miR-137 may be involved in two main pathogenic pathways of SONFH, there is no report concerning the application of miR-137 in the treatment of SONFH.

In the present study, a rat model of SONFH was established. The expression of miR-137-3p and Runx2 in the femoral head and SDF-1α levels in serum were evaluated. Furthermore, the interactions between rat (rno)-miR-137-3p and Runx2 or CXCL12 were verified. Following that, the effects of miR-137-3p silencing on osteogenesis and angiogenesis were investigated *in vitro*. Finally, miR-137-3p-silenced BMSCs were transplanted into SONFH rats, and the preventative effects of miR-137-3p-silenced BMSCs were examined *in vivo*. The related mechanisms of this protection were also explored.

## Materials and Methods

### Radiographic monitoring and expression of miR-137-3p, Runx2 and SDF-1α in a rat SONFH model

**Figure 1:**
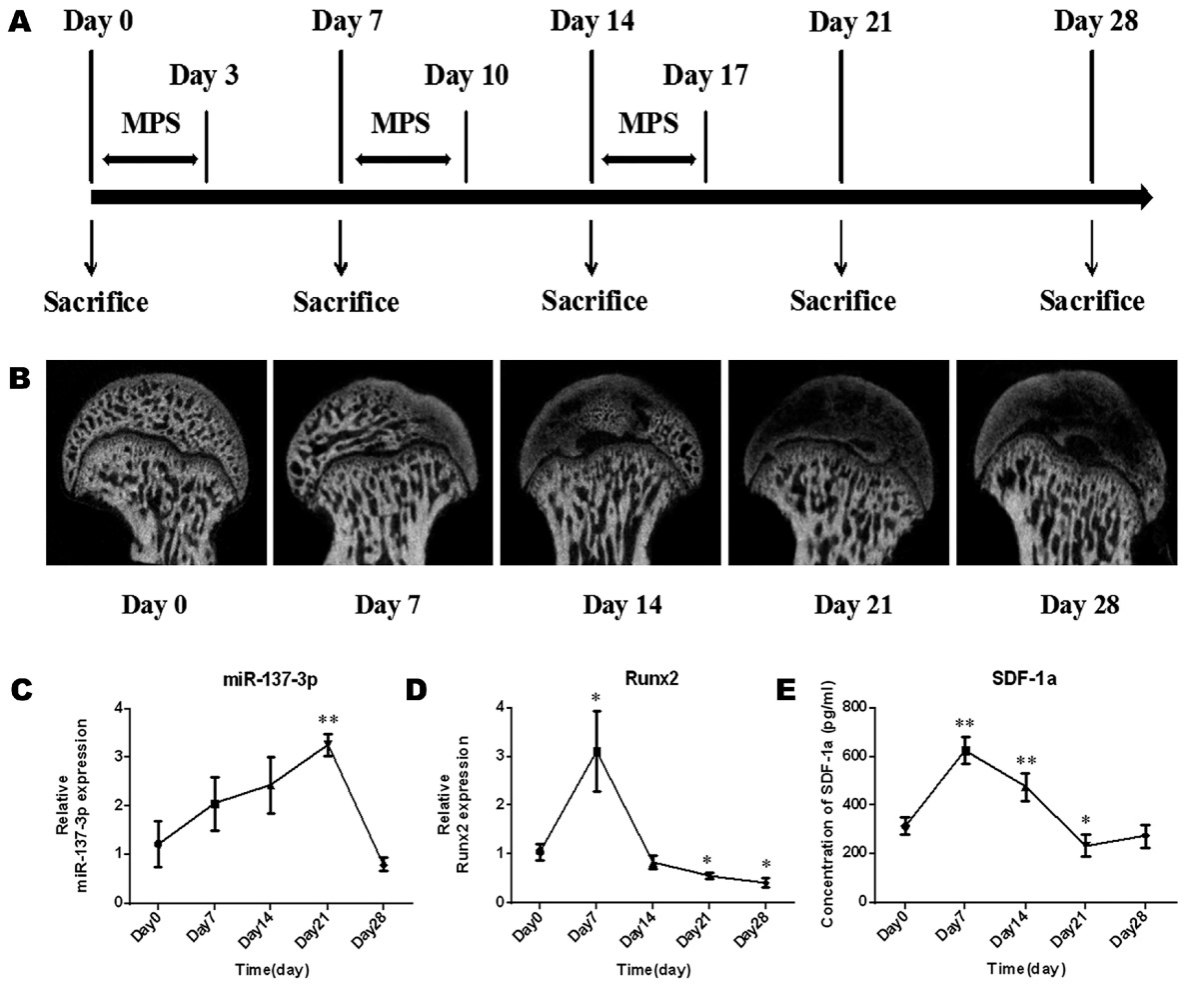
** Micro-CT monitoring and expression changes of miR-137-3p, Runx2 and SDF-1α in the process of SONFH model establishment. (A)** The protocols used to establish the SONFH model and to collect samples are illustrated. **(B)** Micro-CT monitoring was performed before or after MPS intervention. **(C & D)** Tissue miR-137-3p and Runx2 expression was detected by qRT-PCR. U6 was used as the internal reference of miR-137-3p. GAPDH was used as the internal reference of Runx2. (E) Serum SDF-1α content was measured by ELISA. All values are presented as the mean ± SD, *P < 0.05 and **P < 0.01.

The model establishment process was performed as previously described and is illustrated in Figure 1A. Briefly, specific pathogen-free (SPF) male Sprague-Dawley (SD) rats (weight, 250 g **±** 20 g) (n = 15) were intramuscularly injected with methylprednisolone (MPS; Pfizer, Shanghai, China) (20 mg/kg/d) for 3 consecutive days per week for 3 weeks. During the process of model establishment, the morphology of the femoral head, the gene expression of miR-137-3p and Runx2 in the femoral head, and the level of SDF-1α in serum were examined at five time points (Figure 1A). In brief, micro-CT scanning was employed to examine morphological changes of the femoral head during the process. Total RNA was extracted from the femoral head using TRIzol (Invitrogen, Carlsbad, CA, USA). Thereafter, reverse transcription of miR-137-3p was performed by the stem-loop method, while quantitative real-time polymerase chain reaction (qRT-PCR) of miR-137-3p was performed using a SYBR Green PCR Master Mix Kit (EZ Bioscience, Beijing, China). U6 was used as the reference gene of miR-137-3p. The primer sequences (BioTNT, Shanghai, China) are listed in Table S1. The relative expression of miR-137-3p was calculated by the 2^-△△Ct^ method. The detection methods of Runx2 and SDF-1α were as described in the “qRT-PCR” and “ELISA” sections, respectively.

### RNA oligos, constructs and antibodies

The rno-miR-137-3p mimics (sequence: 5′-UUAUUGCUUAAGAAUACGCGUAG-3′), mimics negative control (NC; sequence: 5′-UUGUACUACACAAAAGUACUG-3′), rno-miR-137-3p inhibitor (sequence: 5′-CUACGCGUAUUCUUAAGCAAUAA-3′) and inhibitor NC (sequence: 5′-CAGUACUUUUGUGUAGUACAA-3′) were synthesized by GenePharma (Shanghai, China). The constructs, including pmirGLO-wt-Runx2, pmirGLO-mt-Runx2, pmirGLO-wt-CXCL12, pmirGLO-mt-CXCL12, pmirGLO-Runx2-PC, pmirGLO-CXCL12-PC, pcDNA3.1-Runx2, pcDNA3.1-CXCL12 and lentiviral vector pEZX-MR03 were also obtained from GenePharma.

The antibodies used for western blotting in our study were: anti-Runx2 (Abcam, Cambridge, UK, 1:1000), anti-CXCL12 (Abcam, 1:1000), anti-β-actin (Sigma-Aldrich, St Louis, MO, USA, 1:2000), and rabbit secondary antibody (Biosynthesis Biotechnology, Beijing, China, 1:5000). The antibodies used for immunohistochemistry were: anti-Runx2 (Abcam, 1:200), type I collagen (COL I; Abcam, 1:200), VEGF (Abcam, 1:200), and SDF-1α (Abcam, 1:150).

### Dual luciferase assay

Recombinant vectors and two positive control (PC) vectors were constructed: pmirGLO-wt-Runx2, pmirGLO-mt-Runx2, pmirGLO-wt-CXCL12, pmirGLO-mt-CXCL12, pmirGLO-Runx2-PC and pmirGLO-CXCL12-PC. HEK293 cells were co-transfected with the miRNAs (miR-137-3p mimics or mimics NC) and reporter vectors (wild-type, mutant-type or PC) using the GP-transfect-mate reagent (GenePharma). A dual luciferase assay kit (Promega, Madison, WI, USA) was employed to detect luciferase activity, according to the manufacturer's protocol.

### Western blot analysis

Protein was extracted from cells using Radio Immunoprecipitation Assay (RIPA) lysis buffer (Beyotime, Guangzhou, China). Protein concentration was determined using the bicinchoninic acid (BCA) (Solarbio, Beijing, China) method. Equal amounts of protein were separated by 10-15% sodium dodecyl sulfate polyacrylamide gel electrophoresis (SDS-PAGE) and then transferred to a polyvinylidene difluoride (PVDF) membrane (Millipore, Billerica, MA, USA). The membrane was blocked with 5% (w/v) non-fat dried milk at room temperature for 1 h. Afterwards, the membrane was incubated with primary antibodies against Runx2, CXCL12 and β-actin at 4℃ overnight followed by incubating with secondary antibodies at 37℃ for 1 h. Thereafter, the proteins were visualized using enhanced chemiluminescence (ECL; Beyotime).

### Isolation, culture and transient transfection of rat BMSCs

BMSCs were isolated from 6-week-old SD rats, which were provided by the Chinese Academy of Sciences. The isolation procedure was performed as described in previous studies 30. After isolation, cells were maintained in α-minimum essential medium (α-MEM; Hyclone, Logan, UT, USA), supplemented with 10% (v/v) fetal bovine serum (FBS; Gibco, Carlsbad, CA, USA) and 1% penicillin-streptomycin (Beyotime) in a humidified atmosphere of 5% CO_2_ at 37°C. Cells were passaged when they reached 80-90% confluence. After three passages, the cells were collected for further experiments. Third-passage or fourth-passage BMSCs were transfected with miR-137-3p mimics, mimics NC, inhibitor and inhibitor NC using Lipofectamine 3000 Reagent (Invitrogen). BMSCs treated with 10 μM dexamethasone (DEX; Solarbio) and BMSCs without any treatment were termed the model and control, respectively.

### HUVEC culture and transfection

Human umbilical vein endothelial cells (HUVECs) were purchased from ScienCell Corporation (Shanghai, China) and cultured in endothelial cell medium (ECM; ScienCell) containing 5% (v/v) fetal bovine serum (ScienCell), 1% (v/v) endothelial cell growth supplement (ECGS; ScienCell) and 1% penicillin-streptomycin (ScienCell) in a humidified atmosphere of 5% CO_2_ at 37°C. Transfection of HUVECs was performed using Lipofectamine RNAiMAX Reagent (Invitrogen) in accordance with the manufacturer's protocol. HUVECs treated with 10 μM DEX (Solarbio) and HUVECs without any treatment were termed as the model and control, respectively.

### Cell viability assay

Cell viability was assessed using a cell counting kit-8 (CCK-8; Dojindo, Kumamoto, Japan), according to the manufacturer's instructions. Aliquots containing 5 × 10^3^ BMSCs (control, transfected mimics or transfected inhibitor) or HUVECs (control, transfected mimics or transfected inhibitor) were seeded into 96-well plates and cultured for 72 h. At the 24 h and 72 h time points, cells were incubated with CCK-8 for a further 2 h, after which the optical density (OD) values were measured at 450 nm using a microplate reader (BioTek, Winooski, VT, USA).

### BMSC differentiation and detection

#### ALP and alizarin red staining

BMSCs were seeded into a 24-well plate at 5 × 10^4^ cells per well. When they reached 60-70% confluence, α-MEM complete medium was discarded, the cell layer was washed twice with phosphate-buffered saline (PBS; Solarbio) and osteogenic differentiation induction medium (Cyagen, Guangzhou, China) was added. The culture medium was replaced every three days. After 7 days of osteogenic induction, the osteogenic differentiation induction medium was discarded, and the cell layer was washed twice with PBS followed by fixation using 4% neutral-buffered formalin. ALP activity was measured using a 5-bromo-4-chloro-3-indolyl phosphate/nitroblue tetrazolium chloride (BCIP/NBT) alkaline phosphatase color development kit (Solarbio), according to the manufacturer's protocol. The staining results were observed under a light microscope (Olympus IX 70, Tokyo, Japan), and the area of staining was evaluated using Image J software (NIH, Bethesda, MD, USA). After 14 days of osteogenic induction, alizarin red staining was performed to evaluate calcium deposits according to the manufacturer's protocol (Cyagen). Then the staining results were observed under a light microscope (Olympus IX 70). To quantify the mineralization, alizarin red was eluted from the monolayer with 10% cetylpyridinium chloride (CPC; Sigma), and the absorbance was measured at 570 nm using a microplate reader (BioTek).

#### Oil red O staining

For adipogenic differentiation, 1 × 10^5^ BMSCs were seeded into a 24-well plate and cultured with adipogenic differentiation induction medium (Cyagen) for 3 weeks, according to the manufacturer's instructions. After fixation of cells by 4% neutral-buffered formalin, oil red O staining was employed to detect lipid droplets. Images were captured using a light microscope (Olympus IX 70), and the content of lipid droplets was assessed using Image J software.

### Angiogenic experiments

#### Transwell assay

Cell migration capacity was evaluated using Transwell chambers (Corning, New York, USA). HUVECs (1 × 10^4^ in 200 μL ECM supplemented with 1% FBS) were loaded into the upper chamber, which was inserted into a 24-well plate with 500 μL of complete medium in the well below. After 24 h of culture, cells which had migrated from the upper layer to the lower layer were fixed with 4% neutral-buffered formalin and stained with 0.1% crystal violet (Solarbio). Results were observed under a microscope (Olympus IX 70). The migration activity was quantified by counting the migrated cells.

#### Wound healing assay

HUVECs were seeded into 6-well plates at 2 × 10^5^ cells per well. After cells reached 100% confluence, the medium was discarded and replaced with serum-free medium and cells were starved overnight. A 1,000 μL pipette tip was used to make a straight scratch across the middle of each well. The cellular debris was rinsed away with PBS and the cells were maintained in serum-free medium. At the time points of 0, 12 and 24 h, photographic images of each plate were acquired at the identical location under a microscope (Olympus IX 70). The distance migrated was assessed using Image J software.

#### Tube formation assay

To perform the tube formation assay, 24-well plates were pre-coated with 200 μL Matrigel (Becton Dickinson, Bedford, USA). HUVECs (1 × 10^5^ cells per well) were then seeded into the plates and cultured with complete medium for 24 h. Capillary-like structures were observed under a microscope (Olympus IX 70) and the capacity of tube formation was quantified by calculating the amount of tubes per field.

### qRT-PCR

Total RNA was extracted from cells using an RNA Purification Kit (EZ Bioscience) in accordance with the manufacturer's protocol, followed by measurement of RNA concentration. For reverse transcription mRNAs of ALP, Runx2, CCAAT/enhancer binding protein α (C/EBPα), CD31 and VEGF, cDNA was synthesized from 1 μg of total RNA using a color reverse transcription kit (EZ Bioscience). Then quantitative PCR was performed using a SYBR Green PCR master mix kit (EZ Bioscience). GAPDH was used as the reference gene, and the primer sequences (BioTNT) of the genes above are listed in Table S1. The following cycling conditions were utilized for RT-PCR: 95℃ for 5 min, followed by 40 cycles at 95℃ for 10 s and 60℃ for 30 s. The relative expression of mRNAs was calculated by the 2^-△△Ct^ method.

### Rescue assays

DEX-treated BMSCs or HUVECs were separately transfected with pcDNA3.1-Runx2 or pcDNA3.1-CXCL12, miR-137-3p mimics, or co-transfected with the constructs and miR-137-3p mimics, whereas cells without any treatment served as control. ALP staining and oil red O staining were performed after BMSCs were differentiated under osteogenic or adipogenic conditions, respectively. The tube formation assay of HUVECs was employed to examine the angiogenic ability *in vitro*. Thereafter, the mRNA expression levels of ALP, C/EBPα and VEGF were analyzed by qRT-PCR.

### Stable cell transfection, animal model establishment and grouping

To achieve stable inhibition of miR-137-3p, lentiviral transfection of BMSCs was performed at a multiplicity of infection (MOI) of 25 in the presence of 6 mg/mL of polybrene (Sigma) with lentiviral vector, pEZX-MR03, miR-137-3p inhibitor constructs, or with empty vectors (pEZX), as reported in a previous study 31. After selection with puromycin (Sigma), stable cell lines were generated and used in further animal experiments.

Forty SPF male SD rats (weight, 250 g **±** 20 g) were obtained from the Chinese Science Academy. The rats were divided into four groups (n = 10 per group): control, model, miR-137-3p silencing and NC. The model establishment process is illustrated in Figure 1A. A number of 1 × 10^7^ lentiviral-transfected miR-137-3p-silenced BMSCs or NC-BMSCs were administered by intravenous injection into rats of the experimental group or NC group at the first week of model establishment; control group rats received only saline. All animal treatment procedures were approved by the Animal Care and Use Committee of Shanghai Jiao Tong University Affiliated Sixth People's Hospital.

### Enzyme-linked immunosorbent assay (ELISA) analysis

To acquire serum, the circulating whole blood of rats was collected from the heart and immediately centrifuged at 600 × *g* for 10 min. Samples of serum or medium were stored at -80℃ for further use. SDF-1α content was measured using an SDF-1α ELISA kit (R&D systems, Minneapolis, MN, USA), according to the manufacturer's instructions. Data are expressed as the relative expression level *in vitro* or picograms per milliliter (pg/mL) *in vivo*.

### Flow cytometry

Peripheral blood mononuclear cells (PBMCs) were separated from sodium heparin-buffered peripheral blood using Ficoll density gradient centrifugation (Solarbio) according to the manufacturer's protocol. Purified PBMCs were resuspended in 0.2 mL of PBS. Then they were incubated with the conjugated antibodies CD45-allophycocyanin (APC) (Bioss, Beijing, China), CD34-fluorescein isothiocyanate (FITC) (Bioss) and VEGFR2-phycoerythrin (PE) (Bioss) at room temperature for 20 min. After appropriate gating with lymphocytes, CD45^low^/CD34^+^/VEGFR2^+^ cells were identified as the EPCs by Fluorescence Activating Cell Sorter (FACS), using a FACSCalibur Flow Cytometer (BD Biosciences, Franklin Lakes, NJ, USA). The absolute number of circulating EPCs was converted and expressed as the number of cells per 100,000 events of PBMCs.

### Microfil perfusion and micro-CT scanning

Cardiac Microfil (Flow Tech, Carver, MA, USA) perfusion of rats was performed under general anesthesia, then the rats were placed at 4℃ for 24 h. After perfusion, the femoral heads were collected, fixed with 4% neutral-buffered formalin at room temperature for 48 h and decalcified using ethylene diamine tetraacetic acid (EDTA) for 3 weeks. The processed femoral head samples were then scanned with a micro-CT (Bruker, Kontich, Belgium) and spatial images were observed using CTvox software. In addition, the unperfused femoral heads were harvested followed by micro-CT scanning (Bruker), and plane images were acquired using DataViewer software. Quantitative data of the trabecular bone parameters, such as bone volume per tissue volume (BV/TV), trabecular number (Tb.N), trabecular thickness (Tb.Th) and trabecular separation (Tb.Sp) were analyzed by CTAn software.

### Hematoxylin and eosin (H&E) staining and immunohistochemistry (IHC)

The femoral head samples were fixed with 4% neutral-buffered formalin, decalcified with EDTA, embedded in paraffin and sectioned at a thickness of 4 μm in the coronal plane. To observe the trabecular structure, some of the sections were subjected to H&E staining. The bone histomorphometry such as BV/TV and ratios of empty lacunae was further measured. The other sections were subjected to immunohistochemistry to analyze the expression levels of Runx2, COL I, VEGF and SDF-1α. Images were captured using a light microscope (Nikon ECLIPSE80i, Tokyo, Japan). The mean density of protein expression was evaluated by Image J software.

### Statistical analysis

All experiments were repeated at least three times. Data analysis was performed with SPSS 20.0 software (IBM Corp., Armonk, NY, USA). All data are presented as the mean **±** standard deviation (SD). Comparisons between two or more groups were conducted using *t* test or one-way analysis of variance (ANOVA), respectively, and a difference with a two tailed *P*-value less than 0.05 was considered statistically significant.

## Results

### Micro-CT images and changes in the level of miR-137-3p, Runx2 and SDF-1α during the establishment of the rat model of SONFH

The morphology of the femoral head deteriorated after steroid treatment (Figure 1B). Some femoral heads even collapsed. In addition, the expression levels of miR-137-3p increased gently in the early stage and reached a peak approximately on day 21. After that, the expression of miR-137-3p decreased to the starting level (Figure 1C). Conversely, the expression of Runx2 (Figure 1D) and the serum SDF-1α content (Figure 1E) merely showed a sharp increase in the acute phase and then a decrease compared to baseline in the mid-late phase.

### Runx2 and CXCL12 mRNAs are direct targets of miR-137-3p

**Figure 2:**
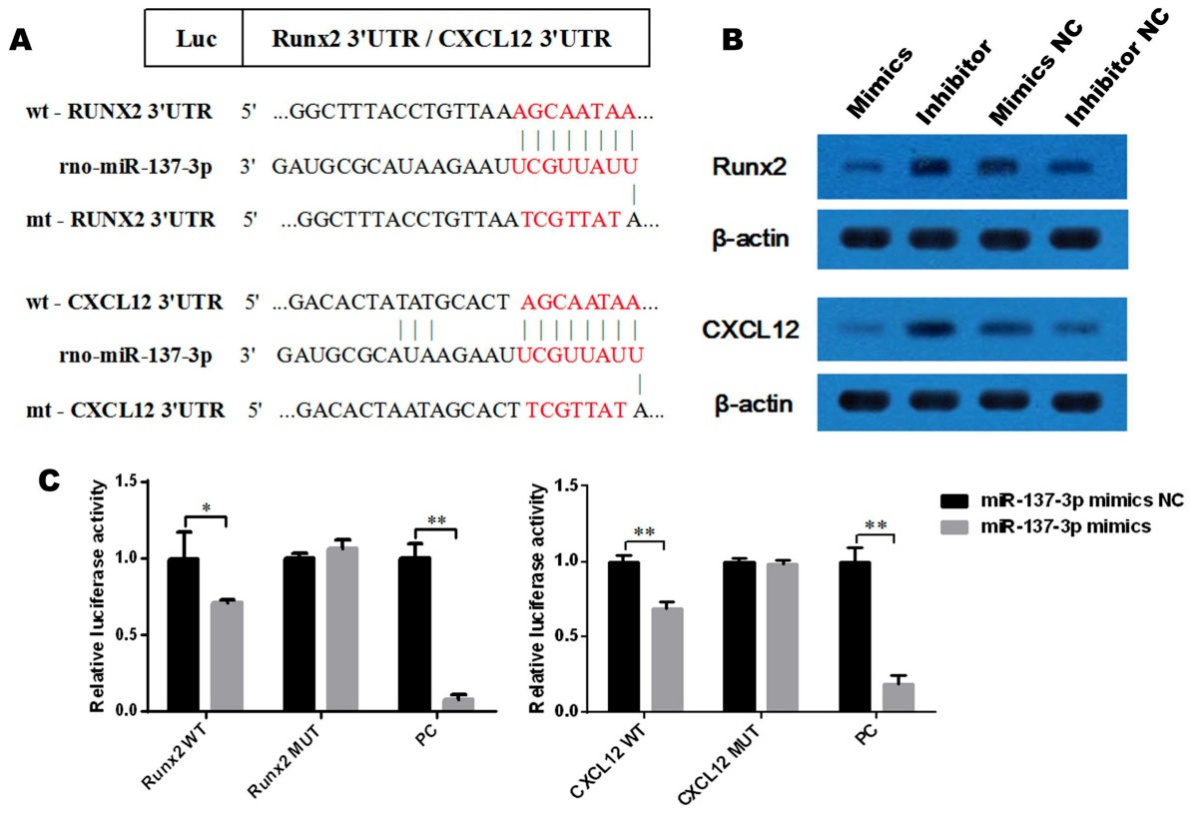
** MiR-137-3p directly targets the 3′UTR of Runx2 and CXCL12. (A)** rno-miR-137-3p seed sequence and the putative binding sequences in the 3′UTR of Runx2 and CXCL12. Both wild type and mutant type sequences were inserted into constructs. **(B)** Expression of Runx2 and CXCL12 at the protein level was detected by western blotting after transfection. β-actin was used as the internal reference. **(C)** HEK293 cells were co-transfected with oligos and constructs. Luciferase activities were examined, and the firefly luciferase activities of each sample were normalized to the Renilla luciferase activities. The values are presented as the mean **±** SD, *P < 0.05 and **P < 0.01.

To determine whether Runx2 and CXCL12 are target genes of miR-137-3p, we first investigated the miRNA databases, such as TargetScan and miRBase, which indicated that the putative binding sequences in the 3′UTR of Runx2 and CXCL12 are a match for the rno-miR-137-3p seed sequence (Figure 2A). Furthermore, the expression of Runx2 and CXCL12 could be suppressed by miR-137-3p at the protein level. Conversely, miR-137-3p inhibitor upregulated the expression of Runx2 and CXCL12 (Figure 2B). In addition, dual luciferase assays revealed that miR-137-3p reduced the luciferase activity of wild type Runx2 and CXCL12 constructs when compared to mutant groups. The positive control confirmed the effectiveness of the method (Figure 2C).

### The effects of miR-137-3p on osteogenesis *in vitro*

**Figure 3:**
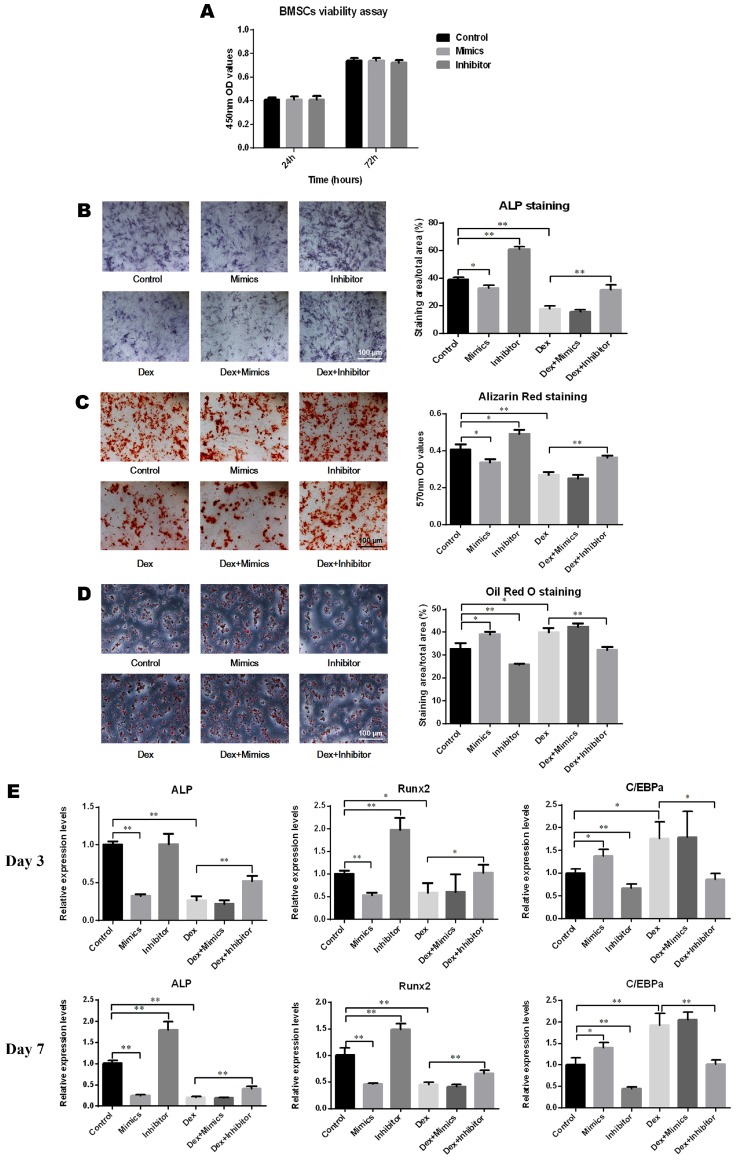
** MiR-137-3p silencing promoted osteogenesis and attenuated adipogenesis of BMSCs *in vitro*. (A)** The impact of oligo transfection on BMSC viability was analyzed by CCK-8 assay. **(B & C)** After induction of osteogenic differentiation, ALP staining (7 d) and alizarin red staining (14 d) were employed to examine ALP activity and calcium deposition respectively, followed by quantitative analysis. **(D)** Oil red O staining and quantitative analysis was performed following adipogenic differentiation of BMSCs. **(E)** After 3 or 7 days of treatment, the expression of the osteogenic markers ALP and Runx2 and the adipogenic marker C/EBPα at the mRNA level was detected by qRT-PCR. GAPDH was adopted as the internal reference. All values are presented as the mean **±** SD, *P < 0.05 and **P < 0.01.

As depicted in Figure 3A, oligo transfection had no significant impact on the cell viability of BMSCs. After induction of osteogenic differentiation, the results of ALP staining demonstrated that the ALP activity increased following transfection with the miR-137-3p inhibitor and decreased with the mimics and DEX. However, this inhibitory effect was significantly reversed by miR-137-3p silencing (Figure 3B). Consistent with this, detection of calcium deposits by alizarin red staining also showed a similar trend to that of ALP activity (Figure 3C). However, oil red O staining indicated that the content of lipid droplets was reduced in the inhibitor group and heightened in the mimics and DEX groups, but miR-137-3p silencing weakened the steroid-induced enhancement of the adipogenic effect (Figure 3D). At the mRNA level, the expression of the osteogenic markers ALP and Runx2 was related to the detection time and exhibited higher levels in the inhibitor groups and lower levels in the mimics groups, compared with the untransfected groups in the presence or absence of DEX after 7 days of treatment (Figure 3E). However, expression of the adipogenic marker C/EBPα showed a diametrically opposite trend (Figure 3E).

### The effects of miR-137-3p on angiogenesis *in vitro*

**Figure 4:**
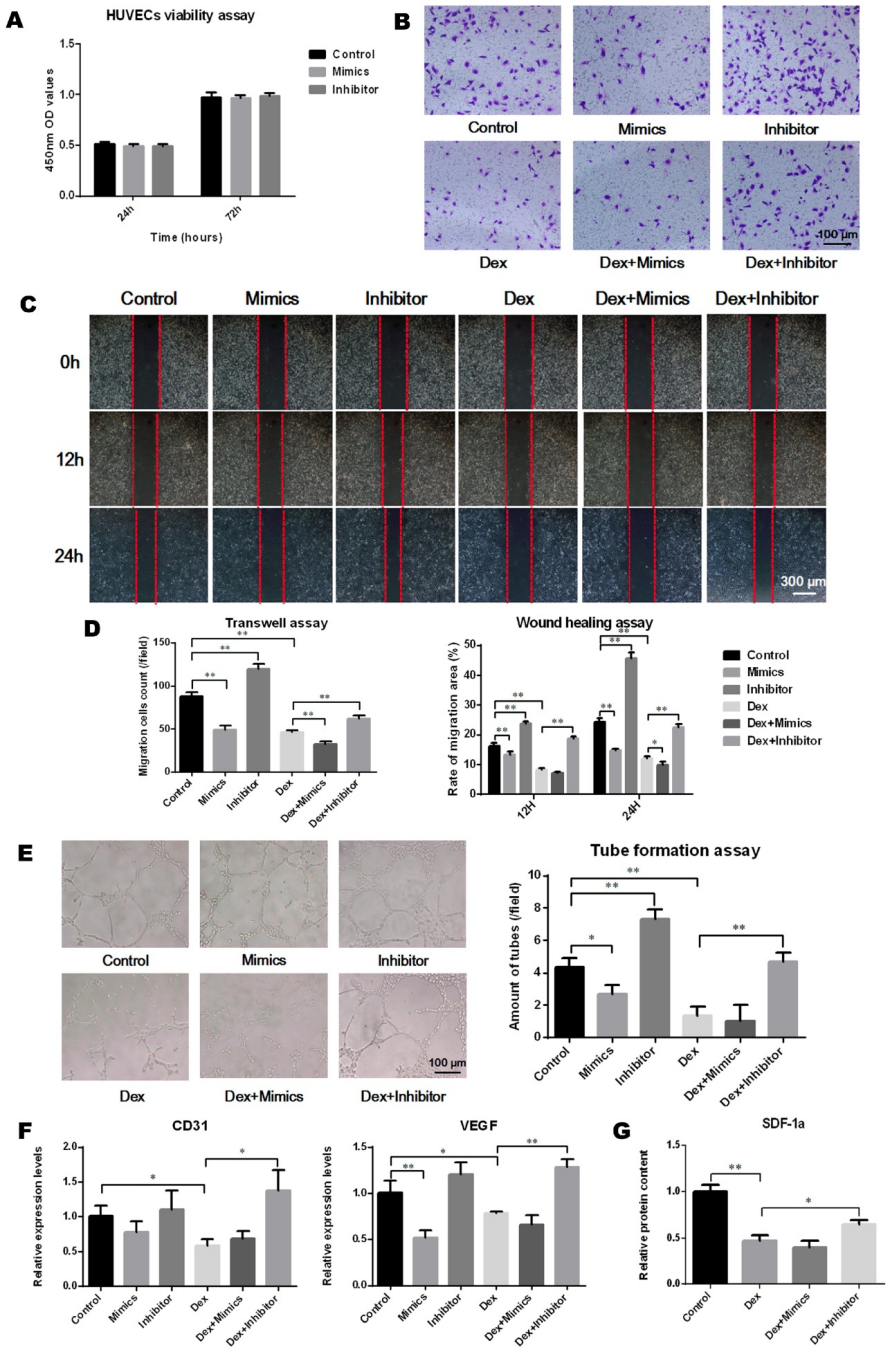
** MiR-137-3p silencing enhanced the angiogenic capacity of HUVECs *in vitro*. (A)** The impact of oligo transfection on HUVEC viability was analyzed by CCK-8 assay. **(B & C)** Transwell assay and wound healing assay were employed to detect the migration capacity changes of HUVECs after treatment. **(D)** Quantitative analysis was performed using Image J software. **(E)** Tube formation assay was used to detect the angiogenic ability of HUVECs and quantitative analysis was performed. **(F)** qRT-PCR results showed the relative expression of CD31 and VEGF. GAPDH was used as the internal reference. **(G)** Relative SDF-1α content in medium was detected by ELISA. All values are presented as the mean **±** SD, *P < 0.05 and **P < 0.01.

The transfection of miR-137-3p had no significant impact on HUVEC viability (Figure 4A). To analyze the effect of miR-137-3p on endothelial cell migration and angiogenesis, *in vitro* experiments were performed. After treatment with DEX, the migration ability of HUVECs was attenuated. However, miR-137-3p silencing significantly restored the cell migration ability impaired by DEX (Figure 4B-D). In line with the results observed in migration assays, miR-137-3p silencing of HUVECs led to obviously higher tube formation capacity, compared with controls. Moreover, both DEX and mimics impaired the tube formation ability of HUVECs, while the inhibitor reversed the steroid-induced effect (Figure 4E). In addition, the qRT-PCR results suggested that the expression of both CD31 and VEGF was augmented in inhibitor groups in the presence of DEX (Figure 4F). Moreover, the SDF-1α content in the medium was significantly reduced after DEX treatment, which was also reversed by silencing of miR-137-3p (Figure 4G).

### Overexpression of Runx2 and CXCL12 without the 3′UTR partially rescued the effects of miR-137-3p

**Figure 5:**
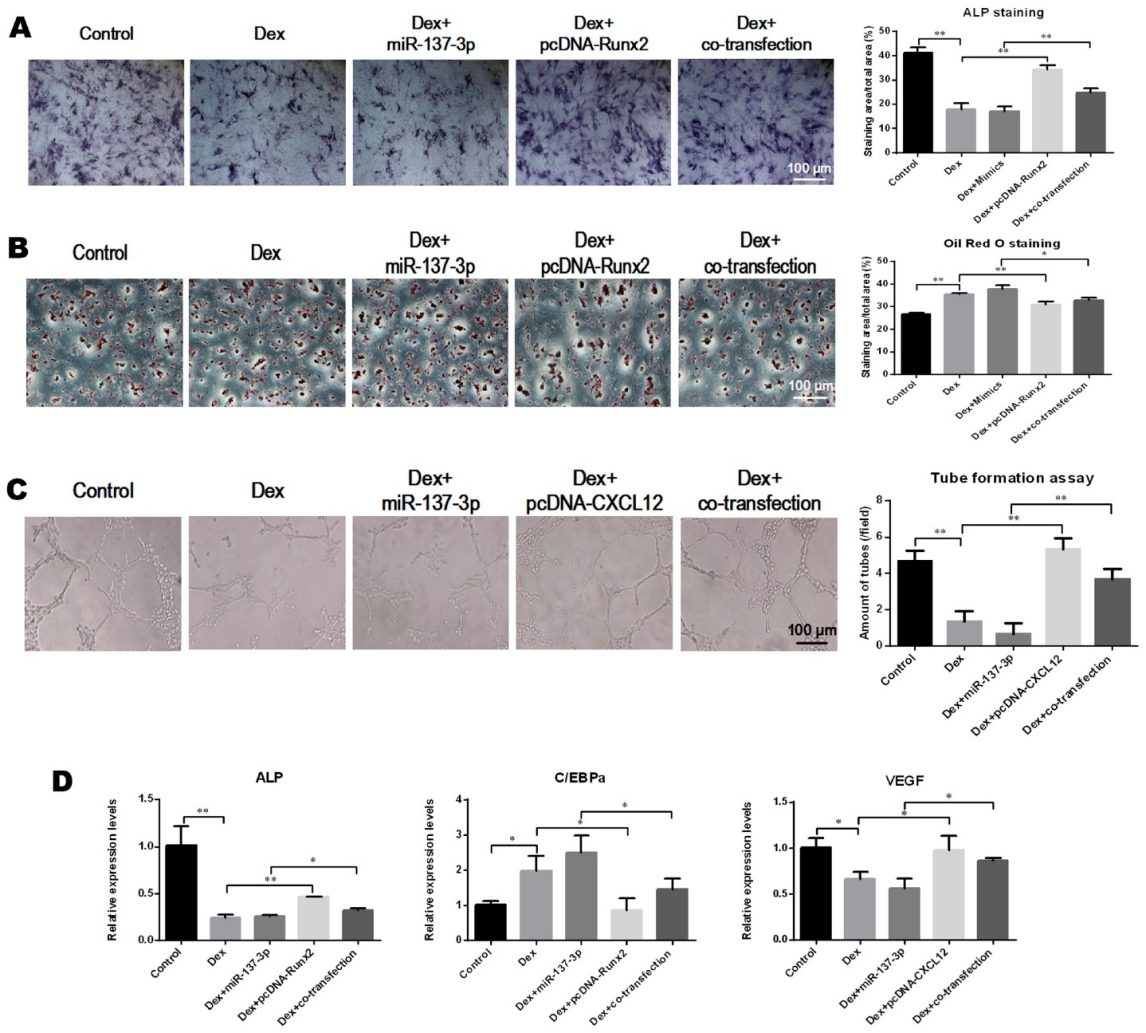
** Overexpression of Runx2 and CXCL12 without the 3′UTR partially rescued the effects of miR-137-3p. (A)** After inducing osteogenic differentiation of BMSCs, ALP staining and quantitative analysis were performed. **(B)** After induction of adipogenic differentiation, lipid droplets were detected by oil red O staining followed by quantitative analysis. **(C)** Tube formation assay and quantitative analysis were employed to illustrate the angiogenic capacity changes of HUVECs. **(D)** The relative expression of ALP, C/EBPα and VEGF at the mRNA level was detected by qRT-PCR. GAPDH was used as the internal reference. All values are presented as the mean **±** SD, *P < 0.05 and **P < 0.01.

The pcDNA 3.1-Runx2 without the 3′UTR, or mimics, or both were transfected into BMSCs. We found that the ALP activity was enhanced in the co-transfection group compared to the group that was transfected with miR-137-3p alone (Figure 5A). However, the formation of lipid droplets in the co-transfection group was reduced compared with the miR-137-3p group in the presence of DEX (Figure 5B). Similarly, pcDNA 3.1-CXCL12 without 3′UTR, or mimics, or both were transfected into HUVECs. The results indicated that tube formation ability was restored in the co-transfection group, compared to the miR-137-3p group in the presence of DEX (Figure 5C). Furthermore, the gene expression of ALP, C/EBPα and VEGF further confirmed that miR-137-3p had inhibitory effects in osteogenesis and angiogenesis, which were partially mediated by down-regulation of Runx2 and CXCL12 (Figure 5D).

### Repair effects of transplantation of miR-137-3p-silenced BMSCs into the rat SONFH model

**Figure 6:**
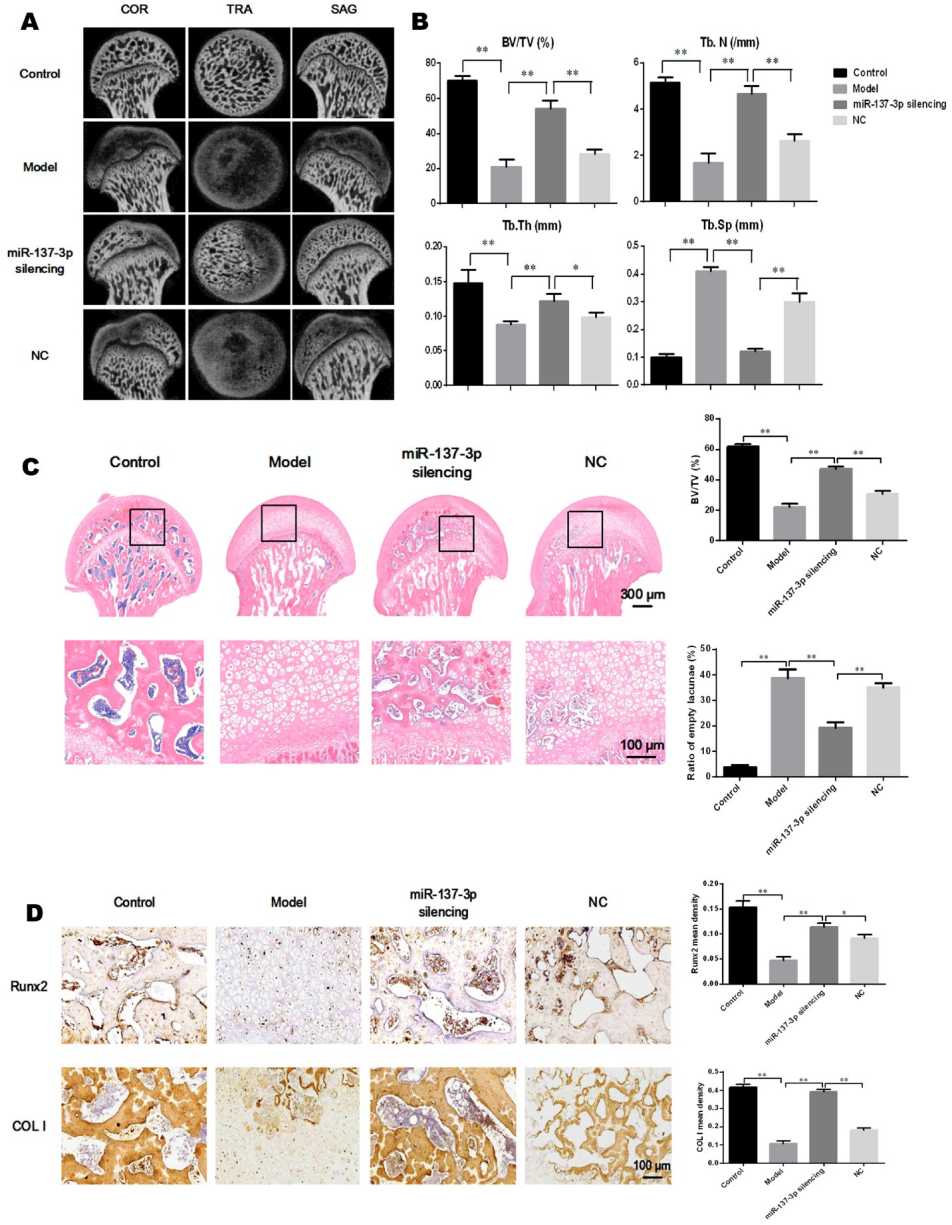
** Transplantation of miR-137-3p-silenced BMSCs enhanced osteogenesis *in vivo*. (A)** Micro-CT scanning images of the femoral head in the coronal plane, transverse plane and sagittal plane were reconstructed in the four groups. **(B)** Evaluation of trabecular parameters including BV/TV, Tb.N, Tb.Th and Tb.Sp based on micro-CT scanning was performed. **(C)** The coronal plane sections stained with H & E were employed to observe the trabecular bone structure of the femoral heads, and the bone histomorphometry, BV/TV and ratios of empty lacunae, was measured. **(D)** Immunohistochemistry images and quantitative analysis showed the local expression levels of Runx2 and COL I. All values are presented as mean **±** SD, *P < 0.05 and **P < 0.01.

The effect of transplantation of miR-137-3p-silenced BMSCs was analyzed in the rat SONFH model. First, the rat model of SONFH was successfully generated, as indicated in Figure 1A. The ideal efficiency of miR-137-3p silencing and BMSCs transplantation was confirmed (Figure S1). The results of micro-CT scanning showed that the NC group only exhibited a slight improvement compared to the model group. By contrast, transplantation of miR-137-3p-silenced BMSCs remarkably attenuated the pathological changes of SONFH (Figure 6A). Furthermore, MPS injection caused serious deterioration of trabecular parameters, such as BV/TV, Tb.N, Tb.Th and Tb.Sp. However, the transplantation of miR-137-3p-silenced BMSCs significantly improved these parameters, while they were only slightly restored in the NC group (Figure 6B). When examined histologically, transplantation of miR-137-3p-silenced BMSCs obviously enhanced osteogenesis in the rat SONFH model. The results of the bone histomorphometry showed that there was more trabecular bone structure and less empty lacunae in the femoral head of the miR-137-3p-silenced group compared to the model or NC groups (Figure 6C). The immunohistochemical and quantitative analysis showed that Runx2 and COL I were downregulated in the model group. The expression of both Runx2 and COL I was significantly restored by the transplantation of miR-137-3p-silenced BMSCs. However, no evidence of improvement was found in the NC group (Figure 6D).

**Figure 7:**
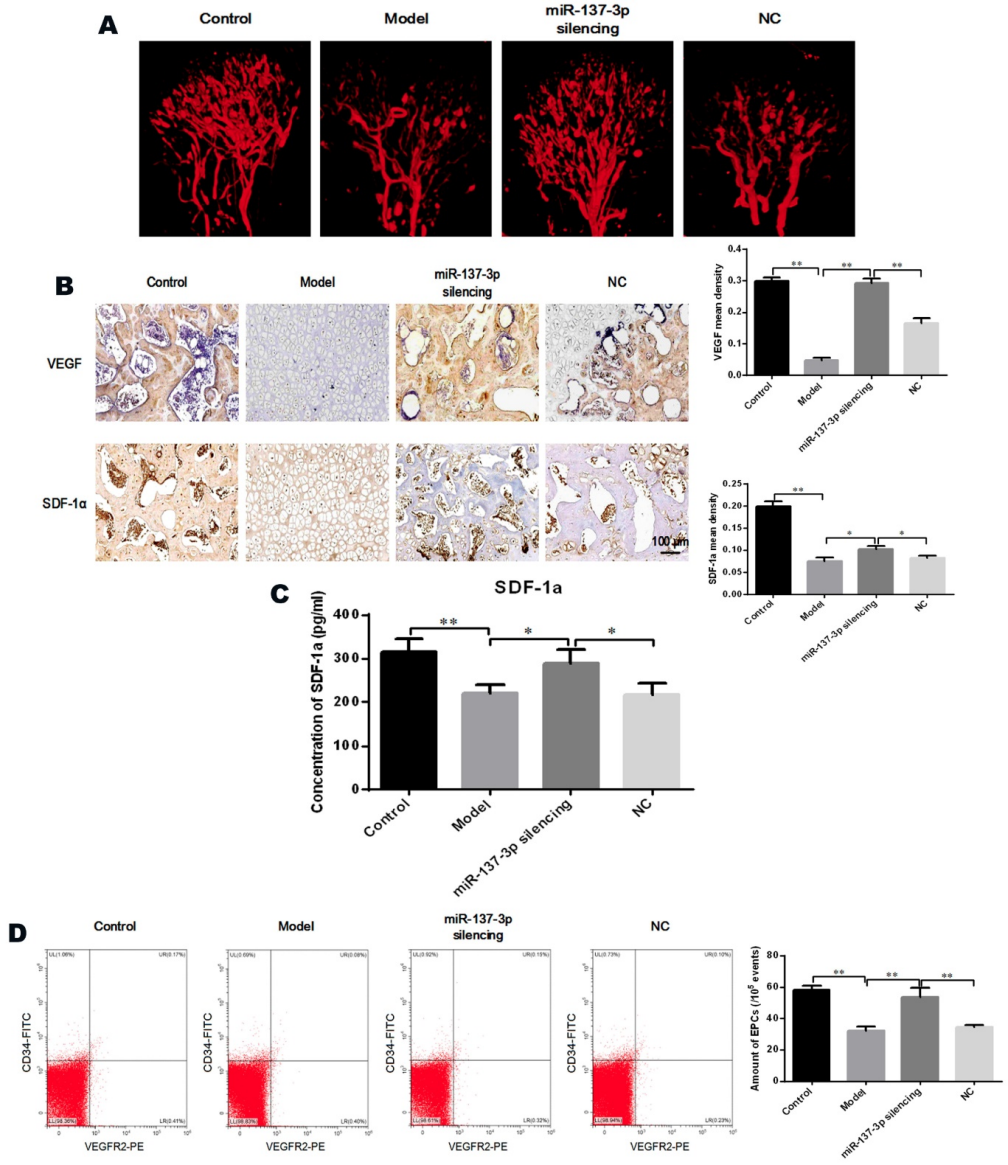
** Transplantation of miR-137-3p-silenced BMSCs promoted angiogenesis *in vivo*. (A)** Local micro-vessel formation was visualized by angiography. **(B)** The local expression of VEGF and SDF-1α was detected by immunohistochemistry, followed by quantitative analysis. **(C)** Serum SDF-1α concentrations in rats in the different groups were analyzed by ELISA. **(D)** The number of circulating EPCs of rats in each group was evaluated by flow cytometry. All values are presented as the mean **±** SD, *P < 0.05 and **P < 0.01.

Angiography was utilized to directly visualize micro-vessels *in vivo*. The images demonstrated that MPS extensively destroyed the micro-vessels of the femoral head (Figure 7A). However, transplantation of miR-137-3p-silenced BMSCs significantly increased micro-vessel volume, compared with the model group or the NC group (Figure 7A). Immunohistochemical and quantitative analysis showed that protein expression of VEGF and SDF-1α was seriously decreased in the model group. However, transplantation of miR-137-3p-silenced BMSCs significantly increased the expression of VEGF and SDF-1α. Meanwhile transplantation of unmodified BMSCs had virtually no effect (Figure 7B). The SDF-1α concentrations in serum were measured by ELISA, the results of which were closely consistent with those of immunohistochemistry (Figure 7C).

### Quantification of EPCs in peripheral blood

The proportion of CD45^low^/CD34^+^/VEGFR2^+^ cells (EPCs) among peripheral blood mononuclear cells was analyzed by flow cytometry. As shown in Figure 7D, the number of EPCs in the circulating blood of model rats was significantly reduced, whereas the decrease was partially reversed by treatment with miR-137-3p-silenced BMSCs. Notably, the number of EPCs was not evidently improved in the NC group, compared to control model rats.

## Discussion

In this study, we observed changes in the levels of miR-137-3p, Runx2 and SDF-1α in a rat model of SONFH, and demonstrated that miR-137-3p is a direct regulator of Runx2 and SDF-1α. Furthermore, the results of the present study showed that miR-137-3p silencing significantly enhanced osteogenesis and angiogenesis *in vitro*. Transplantation of miR-137-3p-silenced BMSCs into the rat SONFH model also achieved a protective effect. To the best of our knowledge, this is the first report of the use of a miRNA for the treatment of SONFH by targeting two genes in two different pathogenic pathways. Silencing of miR-137-3p exhibited ideal repair potential for SONFH.

Previous studies indicated that Runx2 was a potential target of miR-137 by microarray and bioinformatics analysis 20, 32, but there was no further experimental verification. As for CXCL12, *Dong et al.* previously identified that CXCL12 was a target gene of human miR-137 by luciferase reporter assay 22. In the present study, we identified both Runx2 and CXCL12 as direct target genes of rno-miR-137-3p. First, transfection with miR-137-3p mimics reduced the protein expression of Runx2 and CXCL12, while the inhibitor increased their expression levels. Moreover, dual luciferase assays revealed that miR-137-3p directly targeted the sequences in the 3′UTR of Runx2 and CXCL12 mRNA. Finally, rescue assays further demonstrated that the miR-137-3p-induced effects in SONFH were totally or partially mediated by Runx2 and CXCL12. These findings suggested that the up-regulation of Runx2 and CXCL12 both contribute to the repair effects of miR-137-3p silencing.

It is well known that differentiation of BMSCs is crucial to SONFH. A variety of physical, chemical or biological factors have been confirmed to affect the differentiation of BMSCs, such as pulsed electromagnetic fields 33, pravastatin 34, and miR-27a 35. Furthermore, Runx2 is known to be essential in the process of osteogenic differentiation 36 and transdifferentiation 37 of BMSCs. As expected, the miR-137-3p inhibitor promoted an increase in calcium deposits and a reduction in lipid droplet formation in the presence or absence of glucocorticoids. Rescue assays suggested that the Runx2 construct weakened the effects of the mimics. These results indicated that silencing of miR-137-3p facilitated BMSC differentiation into the osteogenic lineage.

Lentiviral transfection of the miR-137-3p inhibitor avoided the disadvantage of transient transfection, which is short-acting. Moreover, SDF-1α-mediated homing of transplanted mesenchymal stem cells to the lesion sites has been verified in our previous study 38 and other study 39, which ensures the homing efficiency of exogenous cells. On the basis of the osteogenic effect of Runx2 36, the effect of transplantation of miR-137-3p-silenced BMSCs in enhancing bone formation was confirmed in SONFH rats. Our data showed that transplantation of miR-137-3p-silenced BMSCs significantly promoted bone regeneration in SONFH rats *in vivo*. However, unmodified BMSCs had an unsatisfactory effect on bone tissue regeneration. Therefore, this obvious difference proved the importance of targeted modification for the repair of SONFH.

The fact that SDF-1α promotes angiogenesis via the CXCL12/CXCR4 or CXCL12/CXCR7 signaling pathways has been widely verified 24, 40, 41. Consistently, miR-137-3p silencing promoted the migration and tube formation of HUVECs in this study, which may be directly related to increased SDF-1α expression. Moreover, previous studies have confirmed that SDF-1α also promotes angiogenesis in wounded and damaged tissues 38, 42. In the current study, animal experiments revealed that transplantation of miR-137-3p-silenced BMSCs enhanced vascularization and angiogenesis in the rat SONFH model, as well as the local or systematic expression of VEGF and SDF-1α. By contrast, unmodified BMSCs had little effect on vascular regeneration. These differences confirmed that the active vascularization process was restored mainly by increased SDF-1α secretion, rather than the natural function of BMSCs. Maintenance of intact vessels is a prerequisite for an adequate blood supply, which is critical to the survival of regenerative bone. All of these findings suggested that the miR-137-3p/SDF-1α axis plays crucial roles in the angiogenesis of SONFH, both *in vitro* and* in vivo*.

A previous study reported that the number of circulating EPCs was dramatically slashed in non-traumatic femoral head necrosis 28. This decrease in the quantity of EPCs may be attributed to the steroid-induced decrease in nitric oxide activity leading to EPC senescence and apoptosis 43, 44. In the process of vascular repair, recent studies have shown that SDF-1α-mediated signaling pathways play important roles in enhancing EPC mobilization and functions in ischemic disease models 23, 24, 45. In our study, systematic and local changes in the expression of SDF-1α and the quantitative variations of EPCs in rat peripheral blood potentially revealed the mechanism of miR-137-3p silencing in promoting micro-vessel repair in SONFH. Increased SDF-1α mobilized more EPCs from the bone marrow to circulating blood where they participate in vascular repair.

Recently, a stronger effect of co-transplantation of BMSCs and EPCs on bone regeneration has been identified 46. In this study, transplantation of miR-137-3p-silenced BMSCs increased SDF-1α secretion and mobilized more EPCs into the circulation. This approach not only achieved a similar effect to co-transplantation, but also overcame the problems that EPCs are difficult to isolate, culture and amplify. Interestingly, a previous study reported that EPCs enhanced the osteogenic capacity of BMSCs by improving their osteogenic microenvironment 47, 48. The bone marrow-derived EPCs in this study not only exerted their functions in vascular repair, but also directly promoted osteogenic differentiation of BMSCs. Therefore, the coupled relationship between osteogenesis and angiogenesis was remarkably reflected in this study, contributing to the satisfactory effects in a mutually-reinforcing manner. However, limitations still exist in this study, in that the details of the increase in the number of circulating EPCs induced by SDF-1α remain unclear, namely whether the CXCL12/CXCR4 or CXCL12/CXCR7 signaling pathway is involved in EPC mobilization. Further study is required to answer this question.

**Figure 8:**
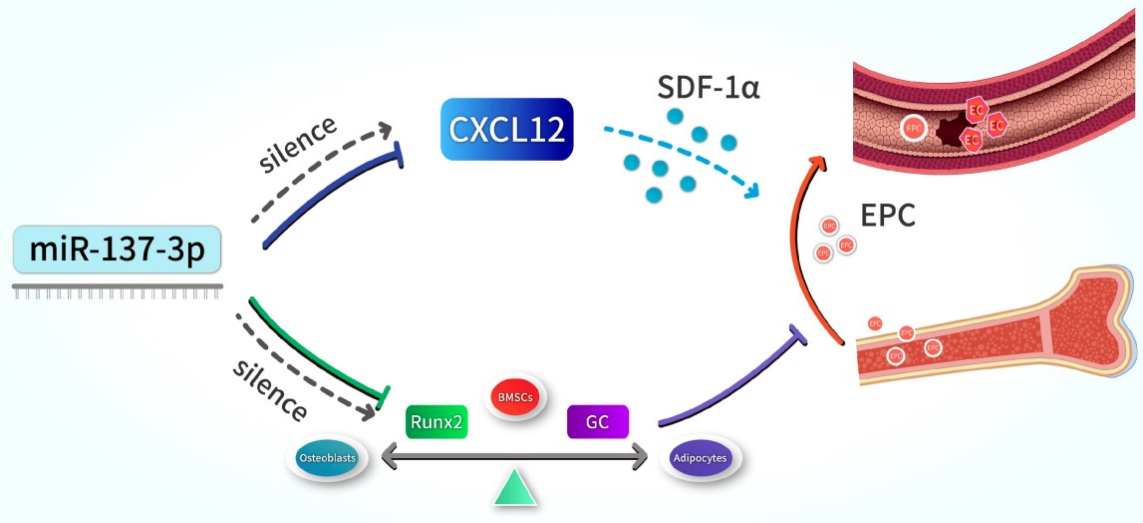
** Schematic illustration.** Silencing of miR-137-3p facilitated osteogenesis in the differentiation balance of BMSCs by enhancing expression of Runx2. It also significantly improved serum SDF-1α content and increased the number of circulating EPCs by up-regulating CXCL12.

In summary, our results indicate that the silencing of miR-137-3p promotes both osteogenesis and angiogenesis *in vitro* and *in vivo*, a mechanism which can be applied in the prevention of steroid-induced femoral head necrosis. In terms of mechanisms, we revealed that miR-137-3p silencing could evidently facilitate osteogenic differentiation and significantly increase the number of circulating EPCs, which can be achieved by up-regulating Runx2 and CXCL12, respectively (Figure 8). All of these findings provide further insights into a potential therapeutic strategy for SONFH.

## Supplementary Material

Supplementary figure and table.

## Abbreviations

SONFH: steroid-induced osteonecrosis of the femoral head
BMSCs: bone marrow-derived mesenchymal stem cells
EPCs: endothelial progenitor cells
miRNAs: microRNAs
miR-137-3p: microRNA-137-3p
ALP: alkaline phosphatase
RUNX2: runt-related transcription factor 2
CXCL12: C-X-C chemokine 12
CXCR4: C-X-C chemokine receptor 4
SDF-1α: stromal cell-derived factor-1α
FGF-2: fibroblast growth factor-2
UTR: untranslated region
DEX: dexamethasone
MPS: methylprednisolone
α-MEM: α-minimum essential medium
FBS: fetal bovine serum
HUVECs: human umbilical vein endothelial cells
CCK-8: cell counting kit-8
OD: optical density
PBS: phosphate-buffered saline
BCA: bicinchoninic acid
qRT-PCR: quantitative real-time polymerase chain reaction
NC: negative control
PC: positive control
COL I: Type I collagen
VEGF: vascular endothelial growth factor
C/EBPα: CCAAT/enhancer binding protein α
GAPDH: glyceraldehyde-3-phosphate dehydrogenase
SDS-PAGE: sodium dodecyl sulfate polyacrylamide gel electrophoresis
PVDF: polyvinylidene difluoride
ECL: enhanced chemiluminescence
MOI: multiplicity of infection
SPF: specific pathogen-free
ELISA: enzyme-linked immunosorbent assay
PBMCs: peripheral blood mononuclear cells
EDTA: ethylene diamine tetraacetic acid
BV/TV: bone volume per tissue volume
Tb.N: trabecular number
Tb.Th: trabecular thickness
Tb.Sp: trabecular separation
H&E: hematoxylin and eosin
IHC: immunohistochemistry
ANOVA: analysis of variance
