# Supplementary material for: Silencing MicroRNA-137-3p, which Targets RUNX2 and CXCL12 Prevents Steroid-induced Osteonecrosis of the Femoral Head by Facilitating Osteogenesis and Angiogenesis
Source: Int J Biol Sci. 2020 Jan 14;16(4):655–70. doi: 10.7150/ijbs.38713 (PMC6990928; doi:10.7150/ijbs.38713)
Supplement: Supplementary file 1 — Supplementary figure and table. [file ijbsv16p0655s1.pdf]

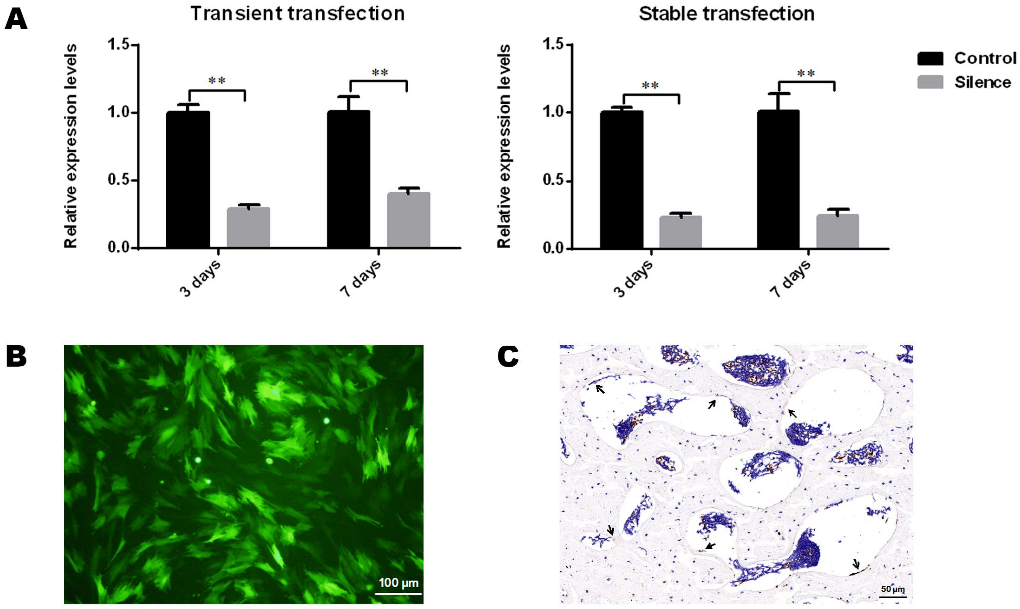


**Figure S1. The efficiency of miR-137-3p silence and BMSC transplantation. (A B)** qRT-PCR results showed that the expression of miR-137-3p was significantly downregulated after both transient and stable transfection on day 3 or 7. **(C)** Lentiviral transfection was employed to acquire GFP-labeled BMSCs. **(D)** The GFP immunohistochemistry of femoral head tissues was performed to examine the homing of BMSCs. The labeled cells were marked by black arrowheads. All values are presented as the mean ± SD, $^{**}P < 0.01$.

**Table S1** The primers sequences used for qRT-PCR.

| Genes | Forword primer sequence (5′-3′) | Reverse primer sequence(5′-3′) |
|---|---|---|
| miR-137-3p | TGCTTAAGAATACGCGTAGC | CAACTGGTGTCGTGGAGT |
| U6 | GCTTCGGCAGCACATAT | TGCGTGTCATCCTTGC |
| Runx2 | ATCATTCAGTGACACCACCA | TTTTGCCTTTAGCCCCTACA |
| ALP | GCTCTGCCGTTGTTTCTCTA | AGATTCCCAAAGCACCTTAT |
| C/EBPα | TCAGACCAGAAGGCTGAGTT | AGGATGAAGACACTGGGACC |
| VEGF | TCAGGAGGACCTTGTGTGATC | CATTGCTCTGTACCTTGGGAA |
| CD31 | CACCGTGATACTGAACAGCAA | GTCACAATCCCACCTTCTGTC |
| GAPDH | TGCCACTCAGAAGACTGTGG | TTCAGCTCTGGGATGACCTT |
